# Histopathological findings associated with prognosis in resected ductal adenocarcinoma of the pancreas

**DOI:** 10.1590/0102-672020260000055e1984

**Published:** 2026-11-09

**Authors:** Eva Valadares SOARES, Eduardo TOAZA, Eduardo CAMBRUZZI

**Affiliations:** 1 Hospital Nossa Senhora da Conceição - Porto Alegre (RS), Brazil.

**Keywords:** Adenocarcinoma, Lymph Node Metastasis, Pancreatic Neoplasms, Pathology, Prognosis, Adenocarcinoma, Metástase Linfática, Neoplasias Pancreáticas, Patologia, Prognóstico

## Abstract

**Background:**

Ductal adenocarcinoma of the pancreas is among the most lethal neoplasms of the digestive tract. It is frequently diagnosed at advanced stages and associated with limited overall survival, even after surgical resection with curative intent. Although pathological staging is fundamental for prognostic stratification, patients with similar anatomical stages may have distinct clinical outcomes, suggesting that additional histopathological factors influence disease evolution.

**Aims:**

This study aimed to assess the prognostic impact of histopathological variables on the overall survival of patients with ductal adenocarcinoma of the pancreas who underwent surgical resection, with emphasis on lymph node involvement and angiolymphatic invasion.

**Methods:**

This observational, retrospective study included patients who underwent surgical resection for ductal adenocarcinoma of the pancreas at a tertiary hospital between 2017 and 2024. Clinical, anatomopathological, and therapeutic data were obtained by reviewing medical records and histopathological reports. Overall survival was estimated using the Kaplan-Meier method, and prognostic factors were analyzed using univariate and multivariate Cox regression models.

**Results:**

A total of 58 patients were included. Lymph node metastases were identified in approximately half of the cases, and angiolymphatic invasion was frequently observed. During follow-up, more than half of the patients died. The median overall survival was 19.63 months. In the univariate analysis, lymph node involvement, lymph node ratio, extranodal extension, and angiolymphatic invasion were associated with worse overall survival. In the multivariate analysis, lymph node status maintained an independent association with worse prognosis, with a progressive increase in risk according to nodal stage, as did angiolymphatic invasion.

**Conclusions:**

Lymph node involvement and angiolymphatic invasion are independent prognostic factors associated with worse overall survival in resected ductal adenocarcinoma of the pancreas. Systematic evaluation of these histopathological variables can support better prognostic stratification, helping individualize postoperative treatment.

## ARTICLE HIGHLIGHTS

• Lymph node involvement and angiolymphatic invasion are independent prognostic factors in resected ductal adenocarcinoma of the pancreas.

• The risk of death increased progressively with advancing pathological lymph node (pN) stage.

• Histopathological variables can be associated with prognostic stratification in resected ductal adenocarcinoma of the pancreas, helping individualize postoperative treatment.

## Summary

**CENTRAL MESSAGE:**

Histopathological findings, particularly angiolymphatic invasion and lymph node involvement, are associated with reduced overall survival in resected ductal adenocarcinoma of the pancreas, reflecting the impact of tumor biology beyond traditional staging.

**PERSPECTIVES:**

The systematic incorporation of qualitative histopathological variables, such as the presence of angiolymphatic invasion and characteristics of lymph node involvement, may improve prognostic stratification of patients with ductal adenocarcinoma of the pancreas. These variables can help identify higher-risk subgroups among patients with similar pathological stages, supporting follow-up and adjuvant treatment individualization. Further studies are needed to validate their application in clinical practice.

## VISUAL ABSTRACT

**Figure:**
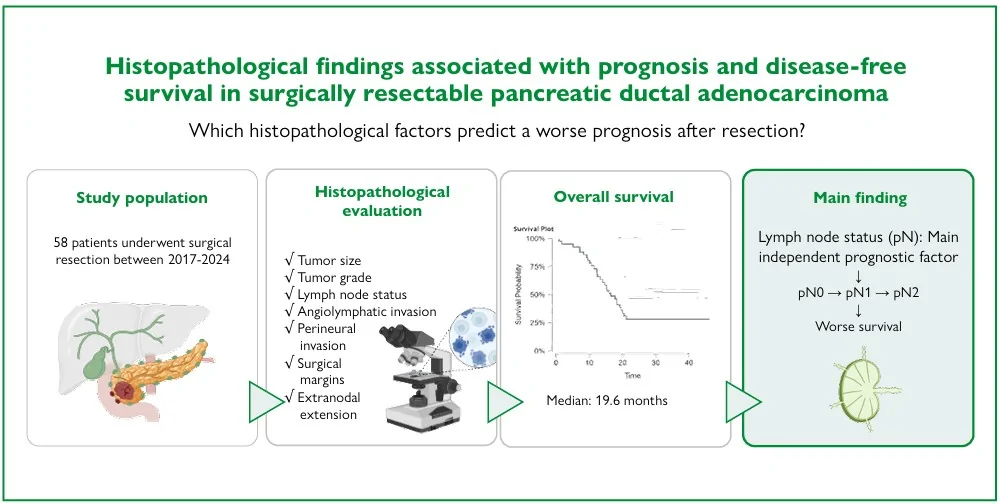
VISUAL ABSTRACT

## RESUMO

**Racional:**

O adenocarcinoma ductal do pâncreas está entre as neoplasias mais letais do trato digestivo. É frequentemente diagnosticado em estádios avançados e associado a uma sobrevida global limitada, mesmo após ressecção cirúrgica com intenção curativa. Embora o estadiamento patológico seja fundamental para a estratificação prognóstica, pacientes com estádios anatômicos semelhantes podem apresentar desfechos clínicos distintos, sugerindo a influência de fatores histopatológicos adicionais na evolução da doença.

**Objetivos:**

Este estudo teve como objetivo avaliar o impacto prognóstico de variáveis histopatológicas na sobrevida global de pacientes com adenocarcinoma ductal do pâncreas submetidos à ressecção cirúrgica, com ênfase no comprometimento linfonodal e na invasão angiolinfática.

**Métodos:**

Estudo observacional e retrospectivo incluiu pacientes submetidos à ressecção cirúrgica por adenocarcinoma ductal do pâncreas em um hospital terciário entre 2017 e 2024. Dados clínicos, anatomopatológicos e terapêuticos foram obtidos por meio da revisão de prontuários e laudos histopatológicos. A sobrevida global foi estimada pelo método de Kaplan-Meier, e os fatores prognósticos foram analisados utilizando modelos de regressão de Cox univariados e multivariados.

**Resultados:**

O total de 58 pacientes foi incluído no estudo. Metástases linfonodais foram identificadas em aproximadamente metade dos casos, e a invasão angiolinfática foi frequentemente observada. Durante o seguimento, mais da metade dos pacientes obituou. A mediana de sobrevida global foi de 19,63 meses. Na análise univariada, o comprometimento linfonodal, a razão linfonodal, a extensão extranodal e a invasão angiolinfática associaram-se a uma pior sobrevida global. Na análise multivariada, o status linfonodal manteve uma associação independente com pior prognóstico, com aumento progressivo do risco conforme o estádio linfonodal, assim como a invasão angiolinfática.

**Conclusões:**

O comprometimento linfonodal e a invasão angiolinfática são fatores prognósticos independentes associados a uma pior sobrevida global no adenocarcinoma ductal do pâncreas ressecado. A avaliação sistemática dessas variáveis histopatológicas pode contribuir para uma estratificação prognóstica mais precisa, auxiliando na individualização do tratamento pós-operatório.

## INTRODUCTION

Pancreatic cancers are among the most lethal tumors of the digestive tract, largely because they are frequently diagnosed at advanced stages^14^ ^,^ ^16^. Overall survival remains limited even when surgical resection is possible, reflecting the biologically aggressive behavior of this tumor subtype^14^ ^,^ ^27^.

Ductal adenocarcinoma of the pancreas is the most frequent histological form of pancreatic cancer and one of the main causes of cancer-related death at this site^5^ ^,^ ^14^. Despite advances in surgical techniques and the progressive incorporation of systemic therapies, oncological outcomes remain unsatisfactory, with high rates of locoregional and distant recurrence after surgical treatment^4^ ^,^ ^18^ ^,^ ^25^ ^,^ ^27^. Surgical resection remains the only therapeutic modality with curative potential^16^ ^,^ ^19^ ^,^ ^23^. However, a significant proportion of patients experience disease progression even after complete resection, suggesting that factors related to tumor biology play a determining role in prognosis beyond the anatomical extent evaluated at the time of diagnosis^7^.

Pathological staging using the TNM (Tumor, Node, and Metastasis) system is a fundamental tool for prognostic stratification of ductal adenocarcinoma of the pancreas. However, it is predominantly based on anatomical criteria^3^ ^,^ ^28^. In clinical practice, patients with similar pathological stages may have different outcomes, underscoring the need to incorporate additional parameters into traditional prognostic assessment^25^ ^,^ ^29^.

Histopathological findings play a central role in this regard. Variables such as tumor differentiation, perineural invasion, angiolymphatic invasion, lymph node involvement, and surgical margin status are routinely evaluated in anatomopathological reports and have been associated with different patterns of recurrence and survival in pancreatic tumors. These parameters provide accessible and reproducible information with direct applications in diagnostic practice^7^ ^,^ ^8^ ^,^ ^9^.

Deoxyribonucleic acid (DNA) repair system investigation through mismatch repair (MMR), although classically associated with other gastrointestinal malignancies, has been explored in ductal adenocarcinoma of the pancreas as a complementary marker^21^. Although deficiency of this system is rare in this tumor subtype, its identification has specific diagnostic and therapeutic implications^10^.

This study aimed to assess the prognostic impact of histopathological variables on resected ductal adenocarcinoma of the pancreas, with emphasis on lymph node involvement and angiolymphatic invasion, as well as their association with overall survival in a retrospective cohort of patients who underwent surgical resection at a tertiary hospital.

## METHODS

An observational, retrospective, analytical study based on a systematic review of electronic medical records and anatomopathological reports of patients who underwent surgical resection for conventional ductal adenocarcinoma of the pancreas at a tertiary hospital in Rio Grande do Sul, Brazil. All cases diagnosed from January 1, 2017, to December 31, 2024, with at least one year of follow-up, were included.

Patients who underwent biopsy only because the disease was unresectable at diagnosis, cases with histological variants other than conventional ductal adenocarcinoma, and non-ductal periampullary tumors were excluded. The final sample consisted of 58 patients who underwent pancreatic resection.

The variables analyzed included age, sex, tumor location, histological grade, tumor size, pathological stage, total number of resected lymph nodes, number of metastatic lymph nodes, lymph node ratio (defined as the ratio of positive lymph nodes to the total number of evaluated lymph nodes), presence of extranodal extension, surgical margin status, presence of angiolymphatic invasion and perineural invasion, and complementary treatment, including neoadjuvant and adjuvant therapy. Extranodal extension was defined as the presence of tumor invasion beyond the lymph node capsule, as described in anatomopathological reports. Surgical margin status was free or compromised according to the original reports.

MMR status, evaluated by immunohistochemistry, was included when available in medical records, with assessment of the expression of MutL homolog 1 gene (MLH1), postmeiotic segregation increased 2 gene (PMS2), MutS homolog 2 gene (MSH2), and MutS homolog 6 gene (MSH6). Cases were classified as proficient (pMMR) or deficient (dMMR).

Overall survival was defined as the interval between the date of surgical resection and the date of death or the last clinical contact recorded in the medical record. Patients who were alive or had no record of death at the last follow-up were considered censored for survival analysis.

Categorical variables were described using absolute and relative frequencies, whereas continuous variables were described using the median and interquartile range. Overall survival was estimated using the Kaplan-Meier method, and comparisons between groups were performed using the log-rank test.

The impact of clinicopathological variables on overall survival was assessed using univariate analysis with Cox regression. Statistically significant or clinically relevant variables were included in a multivariate Cox regression model to identify independent prognostic factors. Results are expressed as hazard ratios (HRs) with their respective 95% confidence intervals (CIs). A statistical significance level of p<0.05 was adopted. Analyses were performed using JASP/Jamovi software.

The research protocol was approved by the Research Ethics Committee at Grupo Hospitalar Conceição and is registered on Plataforma Brasil (CAAE no. 95014625200005530).

## RESULTS

During the period analyzed in the study, 88 patients diagnosed with ductal adenocarcinoma of the pancreas were identified. Of these, 58 underwent surgical resection during the study period and were included in the analysis.

The sample was predominantly composed of women (56.9%). Most tumors were located in the head of the pancreas (87.9%) and had a moderately differentiated histological grade (91.4%), with no cases of well-differentiated adenocarcinoma. Table 1 lists the main clinical, anatomopathological, and therapeutic characteristics.

**Table 1.** Clinical and anatomopathological characteristics of patients with ductal adenocarcinoma of the pancreas who underwent surgical resection (n=58).

Variable	Median (interquartile range)		
Age (years)	64.0 (56.2-70.8)		
Tumor size (cm)	3.2 (2.5-3.9)		
Variable	Group	n	%
Age group (years)	≤60	24	41.4
	>60	34	58.6
Sex	Women	33	56.9
	Men	25	43.1
Tumor location	Head	51	87.9
	Body	4	6.9
	Tail	3	5.2
Degree of differentiation	Well-differentiated	0	0.0
	Moderately differentiated	53	91.4
	Poorly differentiated	5	8.6
pT	pT1	5	8.6
	pT2	43	74.1
	pT3	8	13.8
pN	pN0	29	50.0
	pN1	23	39.7
	pN2	6	10.3
Lymph node metastasis	Absent	29	50.0
	Present	29	50.0
Surgical margin status	Negative (R0)	51	87.9
	Positive (R1)	7	12.1
Perineural invasion	Present	46	79.3
	Absent	12	20.7
Angiolymphatic invasion	Present	41	70.7
	Absent	17	29.3
Extranodal extension	Present	6	10.3
	Absent	52	89.7
Neoadjuvant therapy	Performed	6	10.3
	Not performed	52	89.7
Adjuvant therapy	Performed	36	62.1
	Not performed	22	37.9
Smoking	Yes	23	39.6
	No	14	24.1
	Not reported	21	36.2
Alcohol abuse	Yes	12	20.7
	No	14	24.1
	Not reported	32	55.2
MMR status (n=19)	Proficient (pMMR)	19	100
	Deficient (dMMR)	0	0

pT: pathological tumor stage; pN: pathological lymph node stage; MMR: DNA mismatch repair.

Perineural invasion was present in 46 patients (79.3%), whereas angiolymphatic invasion was observed in 41 cases (70.7%). Lymph node metastases (pN+) were identified in 29 patients (50.0%), being classified as pN1 in 23 cases (39.7%) and pN2 in six cases (10.3%). Extranodal extension was observed in six patients (10.3%). Surgical margins were free of tumor in 51 patients (87.9%), whereas seven cases (12.1%) had involved margins.

MMR system evaluation was performed in 19 patients (32.8%), all of whom were classified as proficient (pMMR). No cases of MMR deficiency (dMMR) were identified.

During the follow-up period, 33 patients (56.9%) died. The median overall survival was 19.63 months, as estimated by the Kaplan-Meier method, with follow-up ranging from 0.95 to 46.15 months.

Kaplan-Meier analysis revealed a significant reduction in overall survival in patients with lymph node metastasis, with a progressively worse prognosis according to pN stage (log-rank test, p<0.05) (Figures 1 and 2). Significant differences in survival were also observed in the presence of angiolymphatic invasion and parameters associated with higher lymph node burden in the unadjusted analysis (Figure 3).

**Figure 1.**
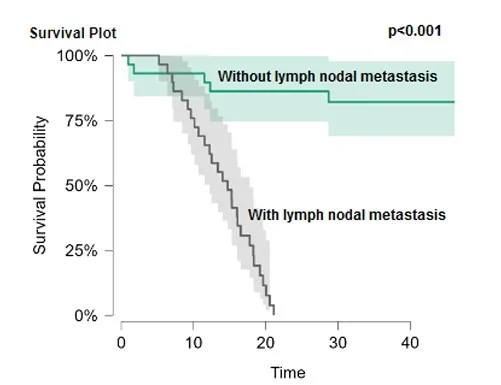
Comparison of Kaplan-Meier curves between patients with and without lymph node metastases.

Source: Personal archive.

**Figure 2.**
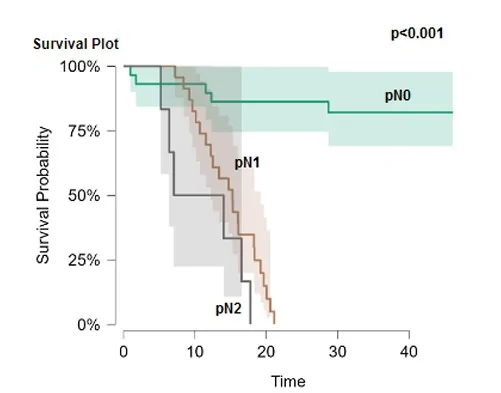
Comparison of Kaplan-Meier curves among patients according to pathological lymph node status. stage.

pN: pathological lymph node status.

Source: Personal archive.

**Figure 3.**
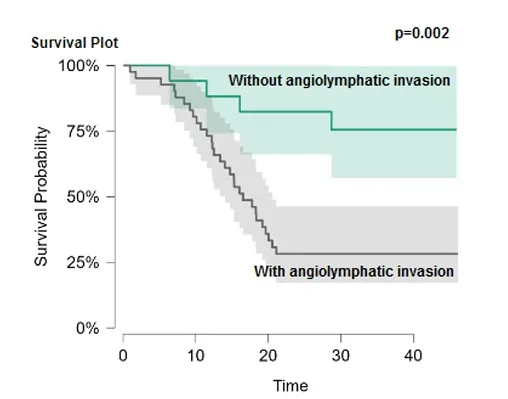
Comparison of Kaplan-Meier curves between patients with and without angiolymphatic invasion.

Source: Personal archive.

In univariate Cox regression analysis, pN status was strongly associated with overall survival. Patients classified as pN1 had a significantly higher risk of death than those classified as pN0 (HR 16.97, 95%CI 5.45-52.79, p<0.001), as did those classified as pN2 (HR 31.21, 95%CI 7.84-124.18, p<0.001). Angiolymphatic invasion was also associated with worse overall survival in the univariate analysis (HR 4.61, 95%CI 1.61-13.21, p=0.004). The other variables, including resection margin status, adjuvant therapy, age, sex, and perineural invasion, were not significantly associated with overall survival.

In multivariate analysis (Table 2), only pN status remained an independent prognostic factor. Both pN1 (adjusted HR 18.75, 95%CI 4.96-70.82, p<0.001) and pN2 (adjusted HR 39.71, 95%CI 8.13-194.09, p<0.001) maintained a significant association with reduced overall survival. Angiolymphatic invasion, surgical margin status, and adjuvant therapy did not maintain an independent association after adjustment. Among patients with lymph node involvement (pN+), the presence of extranodal extension was evaluated exploratorily. There was a tendency toward reduced overall survival in cases with extranodal extension compared with those without this finding, although the difference was not significant (p=0.162).

**Table 2.** Overall survival analysis.

Univariate Cox analysis				
Variable	Comparison	HR	95%CI	p-value
pN	pN1 vs. pN0	16.97	5.45-52.79	<0.001
	pN2 vs. pN0	31.21	7.84-124.18	<0.001
Angiolymphatic invasion	Present vs. absent	4.61	1.61-13.21	0.004
Surgical margin status	R1 vs. R0	1.13	0.39-3.21	0.825
Adjuvant therapy	Yes vs. no	0.68	0.34-1.36	0.272
Age (years)	Continuous	0.98	0.95-1.02	0.291
Sex	Men vs. women	1.40	0.71-2.79	0.332
Perineural invasion	Present vs. absent	1.21	0.50-2.93	0.675
Multivariate analysis				
Variable	Adjusted HR		95%CI	p-value
pN1 (vs. pN0)	18.75		4.96-70.82	<0.001
pN2 (vs. pN0)	39.71		8.13-194.09	<0.001
Angiolymphatic invasion	0.95		0.27-3.41	0.940
Surgical margin status			0.19-1.77	0.341
Adjuvant therapy	0.58		0.32-1.33	0.244

HR: hazard ratio; CI: confidence interval; pN: pathological lymph node stage.

The multivariate analysis was adjusted for clinically relevant variables and those with p<0.10 in the univariate analysis. Pathological lymph node status was included as a categorical variable (pN0, pN1, and pN2).

In multivariate analysis (n=58, 33 events), pN status was independently associated with worse overall survival, with a progressive increase in risk in the pN1 and pN2 categories compared with pN0 (p<0.001). Angiolymphatic invasion, surgical margin status, and adjuvant therapy did not maintain an independent association.

## DISCUSSION

Conventional ductal adenocarcinoma of the pancreas predominantly presents as an infiltrative lesion of the pancreatic head. It is more frequently diagnosed in men, particularly in the presence of risk factors such as chronic pancreatitis, alcohol abuse, and smoking^2^ ^,^ ^3^ ^,^ ^30^. Originating from the ductal epithelium, this malignancy is often diagnosed at advanced stages and characterized by aggressive biological behavior, with large lesions and involvement of peripancreatic tissues^1^ ^,^ ^4^ ^,^ ^5^. The results of the present study reinforce this aggressive profile, evidenced by the short overall survival observed even after surgical resection^6^ ^,^ ^7^. These findings are consistent with previously described clinical series, such as those of Jemal et al.^10^ and Sohn et al.^23^, which demonstrated high mortality and early recurrence despite therapeutic intervention.

Histopathologically, perineural and angiolymphatic invasions were highly frequent, which are characteristics of ductal adenocarcinoma of the pancreas^8^ ^,^ ^9^ ^,^ ^16^. Perineural invasion is recognized as an important mechanism of local tumor dissemination and a marker of biological aggressiveness, as described by Iwasaki et al.^9^ and Nozzoli et al.^19^ However, in the present study, this finding was not significantly associated with overall survival, possibly reflecting limitations related to the small sample size and the retrospective nature of the cohort, without ruling out its relevance as a marker of local aggressiveness.

Angiolymphatic invasion showed a significant association with worse overall survival in the univariate analysis, corroborating its role as a marker of greater potential for tumor dissemination and more aggressive biological behavior. However, this effect was not maintained after multivariate adjustment^12^ ^,^ ^13^ ^,^ ^14^. This lack of significance suggests that its prognostic influence is partially mediated by lymph node involvement, a biologically plausible finding that has been widely described in the literature. Thus, angiolymphatic invasion remains an important histopathological marker of tumor aggressiveness, although it was not an independent prognostic factor in the present cohort^13^ ^,^ ^14^ ^,^ ^15^.

Lymph node spread was identified as the main independent prognostic determinant in the current study. A progressive increase in the risk of death was observed according to the extent of lymph node involvement, reinforcing its central role in the prognostic stratification of ductal adenocarcinoma of the pancreas. These results are consistent with previously published data and support the relevance of a systematic, detailed lymph node evaluation in surgical pathology^15^ ^,^ ^17^ ^,^ ^19^ ^,^ ^20^ ^,^ ^28^.

Extranodal extension has been described as an additional marker of tumor aggressiveness in different solid neoplasms^3^ ^,^ ^11^ ^,^ ^21^ ^,^ ^22^ ^,^ ^23^ ^,^ ^24^. In the present study, its evaluation was limited. It was not possible to include it in the multivariate model due to the limited number of cases. Nevertheless, the observed trend suggests a possible prognostic impact, underscoring the need for investigation in larger series and studies specifically designed for this purpose.

Although the lymph node ratio was significantly associated with overall survival in univariate analysis, it was not included in the final multivariate model because of collinearity with other variables related to lymph node status. This methodological approach aimed to avoid overestimation of prognostic effects and ensure greater stability of the statistical model, prioritizing independent and clinically interpretable variables.

Regarding surgical margin status, most patients underwent resections with negative margins. Although traditionally considered a relevant prognostic factor^1^ ^,^ ^2^ ^,^ ^4^ ^,^ ^19^ ^,^ ^21^, studies such as the one by Verbeke and Gladhaug^26^ suggest that, in ductal adenocarcinoma of the pancreas, the biological aggressiveness of the disease often has a more determining impact on clinical evolution than isolated margin involvement, which may explain tumor recurrence even after resections are considered complete.

Evaluation of the MMR system did not identify cases of deficiency in the study sample. This finding is consistent with previous studies demonstrating a low prevalence of this phenotype in ductal adenocarcinoma of the pancreas^2^ ^,^ ^4^ ^,^ ^21^ ^,^ ^25^. Although rare, the presence of specific molecular alterations may be associated with distinct prognostic subgroups, as described by Schlitter et al.^22^, reinforcing the complementary role of molecular evaluation in characterizing these tumors.

Some limitations should be considered when interpreting the results, including the single-center nature of the study and the relatively limited number of eligible cases. Nevertheless, sample histological homogeneity and systematic analysis of relevant histopathological variables confer consistency and robustness to the findings.

In summary, the results of this study indicate that pN status is the main independent prognostic factor for overall survival in resected ductal adenocarcinoma of the pancreas. Angiolymphatic invasion, although associated with worse prognosis in univariate analysis, appears to reflect a more aggressive tumor phenotype closely related to lymph node involvement. Systematically incorporating these parameters into anatomopathological evaluation may favor better prognostic stratification and help individualize therapeutic strategies.

As summarized in Table 3, the literature showed the prognostic impact of lymph node involvement on ductal adenocarcinoma of the pancreas. Angiolymphatic invasion was less frequently analyzed in isolation but, when evaluated, was associated with worse overall survival, in agreement with the findings of the present study.

**Table 3.** Synthesis of studies that analyzed prognostic histopathological variables in ductal adenocarcinoma of the pancreas.

Author	n	Angiolymphatic invasion	Lymph node metastasis (pN)	Lymph node extension*	Degree of differentiation	Impact on overall survival
Sohn et al.^23^	616	Not reported	Yes (p<0.001)	Not reported	Not reported	Lymph node involvement associated with worse overall survival.
Morita et al.^15^	60	Yes (p<0.01)	Yes (p<0.05)	Not reported	Not reported	Lymphatic invasion as an independent prognostic factor.
Nozzoli et al.^19^	117	Not reported	Yes (p<0.05)	Not reported	Reported	Extensive perineural invasion associated with worse overall survival.
Iwasaki et al.^9^	80	Yes (p<0.05)	Yes (p<0.05)	Not reported	Reported	Neural invasion associated with worse overall survival.
Schlitter et al.^22^	177	Not reported	Yes (p<0.001)	Not reported	G3 (p<0.001)	High grade and pN1 as independent factors.

*Lymph node extension, number of positive lymph nodes and/or lymph node ratio (when explicitly reported).

pN: pathological lymph node status.

## CONCLUSIONS

The findings of this study confirm the aggressive behavior of ductal adenocarcinoma of the pancreas, evidenced by limited overall survival even among patients who underwent surgical resection and adjuvant treatment.

Analysis of histopathological variables showed that pN status was the main independent prognostic factor associated with worse overall survival in the cohort studied, with risk increasing with the extent of lymph node involvement.

Angiolymphatic invasion, although associated with worse prognosis in univariate analysis, appears to reflect a more aggressive tumor phenotype closely related to lymph node involvement and was not an independent factor after multivariate adjustment.

Despite the limitations inherent to the single-center, retrospective study design, the results underscore the central role of surgical pathology in the prognostic stratification of resected ductal adenocarcinoma of the pancreas. Prospective, multicenter studies are needed to validate these findings and explore their applicability to the individualization of therapeutic strategies.

## Data Availability

The datasets generated and/or analyzed during the current study are available from the corresponding author upon reasonable request.

## References

[1] Carvalho LFA, Gryspeerdt F, Ceelen W, Geboes K, Ribeiro S, Hoorens A (2025). Prediction of surgical resectability after FOLFIRINOX chemotherapy for
borderline resectable and locally advanced pancreatic cancer (PeRFormanCe): a
multicenter prospective trial - trial protocol. BMC Surg.

[2] Allen PJ, Kuk D, Del Castillo CF, Basturk O, Wolfgang CL, Cameron JL (2017). Multi-institutional Validation Study of the American Joint Commission
on Cancer (8th Edition) changes for T and N staging in patients with pancreatic
adenocarcinoma. Ann Surg.

[3] Argon A, Öz Ö, Kebat TA (2023). Evaluation and prognostic significance of tumor budding in pancreatic
ductal adenocarcinomas. Indian J Pathol Microbiol.

[4] Bosman FT, Carneiro F, Hruban RH, Theise ND (2019). WHO classification of tumours of the digestive system.

[5] Herman JM, Swartz MJ, Hsu CC, Winter J, Pawlik TM, Sugar E (2008). Analysis of fluorouracil-based adjuvant chemotherapy and radiation
after pancreaticoduodenectomy for ductal adenocarcinoma of the pancreas: results
of a large, prospectively collected database at the Johns Hopkins
Hospital. J Clin Oncol.

[6] Hidalgo M (2010). Pancreatic cancer. N Engl J Med.

[7] Hruban RH, Adsay NV, Albores-Saavedra J, Compton C, Garrett ES, Goodman SN (2001). Pancreatic intraepithelial neoplasia: a new nomenclature and
classification system for pancreatic duct lesions. Am J Surg Pathol.

[8] Ibuki E, Kadota K, Kimura N, Ishikawa R, Oshima M, Okano K (2023). Prognostic significance of tumor budding in patients with pancreatic
invasive ductal carcinoma who received neoadjuvant therapy. Heliyon.

[9] Iwasaki T, Hiraoka N, Ino Y, Nakajima K, Kishi Y, Nara S (2019). Reduction of intrapancreatic neural density in cancer tissue predicts
poorer outcome in pancreatic ductal carcinoma. Cancer Sci.

[10] Jemal A, Ward EM, Johnson CJ, Cronin KA, Ma J, Ryerson B (2017). Annual Report to the Nation on the Status of Cancer, 1975-2014,
featuring survival. J Natl Cancer Inst.

[11] Kayahara M, Nagakawa T, Ohta T, Kitagawa H, Ueno K, Tajima H (1999). Analysis of paraaortic lymph node involvement in pancreatic carcinoma:
a significant indication for surgery?. Cancer.

[12] Kurata Y, Shiraki T, Ichinose M, Kubota K, Imai Y (2021). Effect and limitation of neoadjuvant chemotherapy for pancreatic
ductal adenocarcinoma: consideration from a new perspective. World J Surg Oncol.

[13] Lapshyn H, Bolm L, Kohler I, Werner M, Billmann FG, Bausch D (2017). Histopathological tumor invasion of the mesenterico-portal vein is
characterized by aggressive biology and stromal fibroblast
activation. HPB (Oxford).

[14] Liebig C, Ayala G, Wilks JA, Berger DH, Albo D (2009). Perineural invasion in cancer: a review of the
literature. Cancer.

[15] Morita K, Oshiro H, Mito K, Mieno MN, Tamba-Sakaguchi M, Niki T (2018). Prognostic significance of the degree of lymphatic vessel invasion in
locally advanced, surgically resectable pancreatic head cancer: a single center
experience. Medicine (Baltimore).

[16] Brasil. Ministério da Saúde. Instituto Nacional de Câncer (2022). Estimativa 2023. Incidência de câncer no Brasil.

[17] Neoptolemos JP, Dunn JA, Stocken DD, Almond J, Link K, Beger H (2001). Adjuvant chemoradiotherapy and chemotherapy in resectable pancreatic
cancer: a randomised controlled trial. Lancet.

[18] Neoptolemos JP, Palmer DH, Ghaneh P, Psarelli EE, Valle JW, Halloran CM (2017). Comparison of adjuvant gemcitabine and capecitabine with gemcitabine
monotherapy in patients with resected pancreatic cancer (ESPAC-4): a multicentre,
open-label, randomised, phase 3 trial. Lancet.

[19] Nozzoli F, Catalano M, Messerini L, Cianchi F, Nassini R, De Logu F (2024). Perineural invasion score system and clinical outcomes in resected
pancreatic cancer patients. Pancreatology.

[20] Oettle H, Post S, Neuhaus P, Gellert K, Langrehr J, Ridwelski K (2007). Adjuvant chemotherapy with gemcitabine vs observation in patients
undergoing curative-intent resection of pancreatic cancer: a randomized controlled
trial. JAMA.

[21] Kumar V, Abbas AK, Aster JC (2021). Robbins & Cotran pathologic basis of disease.

[22] Schlitter AM, Segler A, Steiger K, Michalski CW, Jäger C, Konukiewitz B (2017). Molecular, morphological and survival analysis of 177 resected
pancreatic ductal adenocarcinomas (PDACs): identification of prognostic
subtypes. Sci Rep.

[23] Sohn TA, Yeo CJ, Cameron JL, Koniaris L, Kaushal S, Abrams RA (2000). Resected adenocarcinoma of the pancreas-616 patients: results,
outcomes, and prognostic indicators. J Gastrointest Surg.

[24] Song A, Liu F, Wu L, Si X, Zhou Y (2017). Histopathologic tumor invasion of superior mesenteric vein/ portal
vein is a poor prognostic indicator in patients with pancreatic ductal
adenocarcinoma: results from a systematic review and meta-analysis. Oncotarget.

[25] Teske C, Stimpel R, Distler M, Merkel S, Grützmann R, Bolm L (2021). Impact of resection margin status on survival in advanced N stage
pancreatic cancer - a multi-institutional analysis. Langenbecks Arch Surg.

[26] Verbeke CS, Gladhaug IP (2012). Resection margin involvement and tumour origin in pancreatic head
cancer. Br J Surg.

[27] Vincent A, Herman J, Schulick R, Hruban RH, Goggins M (2011). Pancreatic cancer. Lancet.

[28] Weyhe D, Obonyo D, Uslar VN, Stricker I, Tannapfel A (2021). Predictive factors for long-term survival after surgery for pancreatic
ductal adenocarcinoma: making a case for standardized reporting of the resection
margin using certified cancer center data. PLoS One.

[29] Zhao Q, Rashid A, Gong Y, Katz MH, Lee JE, Wolf R (2012). Pathologic complete response to neoadjuvant therapy in patients with
pancreatic ductal adenocarcinoma is associated with a better
prognosis. Ann Diagn Pathol.

[30] Zhou QQ, Ren YM, Shi SM, Liu TF (2025). Microenvironmental code of pancreatic ductal adenocarcinoma: the
prognostic symphony of budding, matrix and lymphocytes. World J Gastrointest Oncol.

